# Consideration of sex/gender in publications of quantitative health-related research: Development and application of an assessment matrix

**DOI:** 10.3389/fpubh.2023.992557

**Published:** 2023-04-04

**Authors:** Sophie Horstmann, Christina Hartig, Ute Kraus, Kerstin Palm, Katharina Jacke, Lisa Dandolo, Alexandra Schneider, Gabriele Bolte

**Affiliations:** ^1^Department of Social Epidemiology, Institute of Public Health and Nursing Research, University of Bremen, Bremen, Germany; ^2^Health Sciences Bremen, University of Bremen, Bremen, Germany; ^3^Helmholtz Zentrum München, German Research Center for Environmental Health, Institute of Epidemiology, Neuherberg, Germany; ^4^Gender and Science Research Unit, Institute of History, Humboldt University of Berlin, Berlin, Germany

**Keywords:** sex, gender, assessment matrix, health research, quantitative research

## Abstract

During the last years the need to integrate sex and gender in health-related research for better and fairer science became increasingly apparent. Various guidelines and checklists were developed to encourage and support researchers in considering the entangled dimensions of sex/gender in their research. However, a tool for the assessment of sex/gender consideration and its visualization is still missing. We aim to fill this gap by introducing an assessment matrix that can be used as a flexible instrument for comprehensively evaluating the sex/gender consideration in quantitative health-related research. The matrix was developed through an iterative and open process based on the interdisciplinary expertise represented in our research team and currently published guidelines. The final matrix consists of 14 different items covering the whole research process and the publication of results. Additionally, we introduced a method to graphically display this evaluation. By developing the matrix, we aim to provide users with a tool to systematically compare sex/gender consideration qualitatively between different publications and even different fields of study. This way, the assessment matrix represents a tool to identify research gaps and a basis for future research. In the long term, the implementation of this tool to evaluate the consideration of sex/gender should contribute to more sex/gender equitable health-related research.

## 1. Introduction

During the last years the need for a theory-based integration of sex and gender into health-related research became increasingly apparent (1–4). Various guidelines are calling for a more systematic inclusion of sex and gender in the entire research process and its reporting (3, 5–7). This includes, in a first step, ensuring the understanding of the terms sex and gender and their appropriate application (7, 8).

In a common understanding, sex refers to biological characteristics related to sexual reproduction such as chromosomes, hormones, and sexual organs (9). The term gender is used to describe a multidimensional social construct that includes identities, norms and relationships. While identities define how individuals see themselves, norms refer to what attitudes and expectations are culturally and socially considered appropriate for the different sex/gender groups. Gender relations explain how people interact with each other on the basis of their sex/gender and how power is distributed between different sex/gender groups. In this way, gender can be viewed on an individual level as well as on a structural and symbolic level (1, 6). Even though often categorized as binary (3, 9), sex and gender are now increasingly arranged on a continuum and understood as fluid. Both sex and gender are assumed to embrace different dimensions that are not interdependent and can therefore be combined in different ways (1). However, sex and gender are considered to be intertwined and might interact with each other (2, 10, 11). Within this publication, we therefore use the term “sex/gender” to indicate this entanglement (12, 13).

Recently, several systematic reviews were conducted to assess the quality of sex/gender consideration within different fields of health-related research (14–18). Nevertheless, within each of these reviews a selective set of criteria was applied which in some cases focused on specific aspects of sex/gender consideration, such as the precise use of terminology (16) or the conceptualization of sex/gender (19). Consequently, despite some overlap, the results and conclusions from particular publications are only partially comparable in terms of their investigation of sex/gender consideration.

Additionally, despite this increasingly intensive analysis of the consideration of sex/gender (14, 15, 18, 20), a matrix for the comprehensive assessment of sex/gender consideration and its visualization is still missing. The standardized visualization of evaluation data is considered a promising opportunity to identify overall patterns and outlying observations (21, 22). It allows the findings of several studies to be compared directly with each other, as they can be displayed in a single diagram (23). A matrix for the evaluation of sex/gender consideration and its visualization would be a good possibility to provide a thorough overview of a publication’s performance, to compare different research areas, and to highlight which criteria are already well met and where further attention is needed.

To fill this gap, we aimed to develop a matrix to assess and visualize the consideration of sex/gender within quantitative health-related studies in a systematic and theory-based way. In contrast to publications which assess the mere consideration of sex/gender in all kind of studies (14, 24), we conducted a more comprehensive evaluation of studies that already analyze the impact of sex/gender in the context of different health related research questions. By introducing the assessment matrix, we aim to provide researchers in the field of quantitative health research with a useful tool to systematically assess a publication’s adherence to current guidelines promoting the adequate consideration of sex/gender in the whole research process.

The matrix was developed and tested within the collaborative research project INGER (integrating gender into environmental health research)^1^ which aims to develop innovative methods for sex/gender-equitable data collection and data analyses in population-based studies on environmental health. One of the project’s objectives was to assess the consideration of sex/gender in different environmental research fields. For this purpose, several systematic reviews were carried out (15, 18). Publications that were identified in two of these systematic reviews were used to exemplary apply the matrix. The matrix is not limited to environmental health research, but applicable in various research fields of quantitative health research.

## 2. Methods

We designed the matrix through an iterative and open process, drawing on the experience we gained during the development of the multidimensional INGER sex/gender concept. The concept was developed from an intersectional perspective to provide a basis for the operationalization of sex/gender in quantitative health research (1). In addition, three published guidelines were in particular included in the matrix development: The Strengthening the Reporting of Observational Studies in Epidemiology (STROBE) statement is a checklist of items that should be included in publications reporting observational research (25). The Sex and Gender Equity in Research (SAGER) guidelines are designed to support authors in the systematic reporting of sex and gender when writing a manuscript (3). The PRISMA-E 2012 statement is an equity extension for The Preferred Reporting Items for Systematic Reviews and Meta-Analysis (PRISMA) statement. It was developed to improve the reporting of equity-focused systematic reviews (26). Since it is assumed that various inequalities in health are caused by sex/gender (27), several of the items of the PRISMA-E 2012 statement were applicable for the development of this matrix. We followed the structure that was implemented with the STROBE guidelines (25) to divide the matrix into the sections title, abstract, introduction, methods, results and discussion. For every section we collected and synthesized recommendations concerning the consideration of sex/gender that we received from published guidelines or that were based on our own expertise.

The assessment items were developed and reviewed through interdisciplinary cooperation of the INGER research group, combining expertise on public health, epidemiology, and gender studies. Two independent authors (S.H. & U.K.) tested the matrix with two different sets of publications. Problems and challenges were discussed and the matrix’ criteria were modified accordingly.

To test the matrix’ application, it was applied to the publications identified within the reviews ‘Sex/Gender Differences in the Association between Residential Green Space and Self-Rated Health—A Sex/Gender-Focused Systematic Review“that was published by Bolte et al. (18) and ‘Sex/Gender-Differences in the Health Effects of Environmental Noise Exposure on Hypertension and Ischemic Heart Disease – A Systematic Review’ by Rompel et al. (15). Regarding the review by Rompel et al. we restricted the assessment using the matrix to the identified studies on the association between environmental noise and hypertension. The search strategies were similar for both reviews with the main inclusion criterion that the studies calculated sex/gender-specific effect estimates. Nevertheless, Bolte et al. only included studies with a clear focus on sex/gender, i.e., at least one keyword for sex/gender had to be mentioned in the publication’s title or abstract, which was not necessary in the review of Rompel et al. Detailed information on the search strategies can be found in the corresponding publications (15, 18).

To visualize the assessment matrix, we used the software program R. The package ‘flextable’ provides a framework for easily creating tables (28). Please see Supplementary material 1 for detailed instructions on the syntax. Nevertheless, the matrix was constructed in such a way that it can easily be created using a simple text editing program. Thus, it can also be applied without advanced software skills.

## 3. Results

The final matrix consists of 14 different items that we consider important when judging the consideration of sex/gender in health-related quantitative studies. The final items relate to the publication’s title and abstract (2 items), introduction (3 items), methods (5 items), results (2 items), and discussion (1 item). One item can be considered as a general principle and therefore applies to the entire publication. Each item consists of at least two different rating levels increasing in quality: The lowest rating level a always means that the criterion has not been fulfilled at all, while the rating levels b to d indicate that the criterion has been met to some degree as explained below.

In the following we explain the 14 items (Table 1) and give examples to illustrate the corresponding criteria in published studies. We want to emphasize that we have selected the text passages presented below from diverse publications because we consider them to be good examples. However, the assessment of particular text passages must not to be mistaken with an overall assessment of the study from which the example was taken, nor does it mean that the examples’ findings or content are reliable. Since in most cases rating level “a: not at all” refers to a lack of sex/gender consideration examples are only given exceptionally.

**Table 1:** Matrix for the assessment of sex/gender consideration in quantitative health-related research.

Criteria	Rating levels
(1) Precise use of sex/gender-specific terminology	Not at all Sex/gender terms are explained and used precisely
**Title and abstract**	
(2) Sex/gender mentioned in the title	Not at all Sex/gender related terms are mentioned in the title
(3) Sex/gender mentioned in the abstract	Not at all Sex/gender is mentioned within the description of the methods and/or the study population in the abstract Presentation of sex/gender findings in the abstract Presentation of sex/gender findings and implications for further practical application or research in the abstract
**Introduction**	
(4) Rationale for the consideration of sex/gender given	Not at all Implicit rationale for the consideration of sex/gender is given Explicit rationale for the consideration of sex/gender is given
(5) Sex/gender mentioned in the objectives	Not at all The analysis of sex/gender is implicitly stated as an objective of the study The analysis of sex/gender is explicitly stated as an objective of the study
(6) Hypotheses given	Not at all Hypothesis of direction of effect are given
**Methods**	
(7) Source of information for recruitment is reported	Not at all Source of sex/gender information for recruitment is reported
(8) Reporting of selection of study population	Not at all All methods of selection of participants are reported. If necessary, all means to ensure adequate representation of sex/gender categories are described (e.g., oversampling)
(9) Data collection and operationalization reported	Not at all The source from which the sex/gender-related information was gathered is reported Source and operationalization of sex/gender-related variables are reported
(10) Consideration of sex/gender variety and multidimensionality	Not at all (binary) Sex/gender is operationalized in three categories (e.g., female, male, divers) Consideration of the variety and/or multidimensionality of sex/gender
(11) Sex/gender-specific analysis	Not at all Sex/gender is only used as a confounder Sex/gender is considered as an independent influencing factor, for subgroup or interaction analysis or a comparable methodology
**Results**	
(12) Description of the study population	Not at all Sex/gender distribution (e.g., proportion or count) of the study population is described
(13) Sex/gender-specific results presented	Not at all Only sex/gender-specific baseline characteristics are reported Only sex/gender-specific effect estimates are reported Baseline characteristics and sex/gender-specific effect estimates are reported
**Discussion**	
(14) Discussion of sex/gender-specific results	Not at all A summary of the sex/gender results of this publication and if appropriate, from other publications is given but not further discussed Sex/gender results of this publication are further discussed against the background of other publications (evidence or theory)

### 3.1. General principles

#### 3.1.1. (1) Precise use of sex/gender-specific terminology (possible rating levels a, b)

To avoid confusion, authors need to report how sex/gender is conceptualized within their publication (5, 6). In this matrix we work with a concept of sex/gender that is currently common in internationally published health-related research (1, 9). However, we acknowledge that there is a variety of understandings of sex/gender in different scientific and cultural contexts. Therefore, in order to receive rating level b, all sex/gender related concepts analyzed within the publication (e.g., sex, gender, femininity) need to be defined and used according to the definitions given in the publication (6, 20). Since journals often set word limitations, there might not always be enough space for a detailed definition within the publication itself. Thus, authors must at least provide an explicit reference to further literature in order to meet the requirements for this criterion. If no definition of the sex/gender related concepts is provided or the introduced terms are not used consistently throughout the reporting of the study, the publication is rated “a: not at all.”

##### 3.1.1.1. Example for imprecise use of terms in the publication title (rating level a)

“*Does sex matter? The influence of gender on gastrointestinal physiology and drug delivery*” (29) (title).

##### 3.1.1.2. Example for a given definition (rating level b)

“*Femininity has been described by Holland as an ‘elusive’ concept, seen variously as a normative order (i.e., a set of psychological traits such as being nurturing), a performance or a process of interaction*.” (30) (p. 2).

Even though we feel that the correct use of terminology is of particular importance, we aim to keep the use of the matrix as simple as possible. Therefore, the remaining criteria are assessed independently of the sex/gender terminology used.

### 3.2. Title and abstract

#### 3.2.1. (2) Sex/gender mentioned in the title (possible rating levels a, b)

To achieve a better integration of sex/gender in health-related research, it is important that studies that provide sex/gender-specific results are visible and can be easily found by other researchers or decision-makers. If publications already indicate in the title that they deal with sex/gender or one specific sex/gender group correct indexing in electronic databases is supported, making studies easier to find *via* search engines (3, 25, 26). This also increases their citation potential which in turn increases the dissemination of sex/gender related research. In order to fulfill rating level b, authors therefore need to use at least on sex/gender keyword within the title. If sex/gender of the study population or the sex/gender aspects considered in the analyses are not given at all in the title, the publication has to be rated with “a: not at all.”

##### 3.2.1.1. Examples for rating level a

“*Environmental exposure to endotoxin and its relation to asthma in school-age children*” (31) (title).

“*Estimating the impact of sustained social participation on depressive symptoms in older adults*” (32) (title).

##### 3.2.1.2. Examples for rating level b

“*Sex/Gender-Differences in the Health Effects of Environmental Noise Exposure on Hypertension and Ischemic Heart Disease—A Systematic Review*” (15) (title).

“*Sexuality and the limits of agency among South African teenage women: Theorising femininities and their connections to HIV risk practises*” (30) (title).

#### 3.2.2. (3) Sex/gender mentioned in the abstract (possible rating levels a, b, c, d)

Since other researchers or decision-makers might only read the abstract to assess a publication’s relevance for their own work, the consideration of sex/gender already needs to be highlighted here (26). A balanced summary of sex/gender related methods and results needs to be given in a short description of the studies’ population and methods (rating level b) as well as the reporting of the study’s findings (rating level c) and implications for further practical application or research (rating level d) (25). Since we consider these levels as increasing in importance, publications should always be rated with the highest level if they meet the requirements. This also applies if the previous rating levels are not met. If sex/gender is not mentioned at all within the abstract, the publication receives the rating “a: not at all.”

##### 3.2.2.1. Example for rating level b

“*Poisson models with natural splines were used to control for time-varying covariates such as season and weather. Separate models were run after stratification by gender, race/ethnicity (White, Hispanic, Black) and education (high school graduation or not)*.” (33) (Without further mentioning of sex/gender in the abstract).

##### 3.2.2.2. Example for rating level c

“*The effect on satisfaction was especially marked among tenants and the presence of recreational values was associated with low or normal body mass index in this group. A less marked positive association with vitality among women was observed. No evident effect on self-rated health was detectable*.” (34) (Without giving sex/gender-related implications for further practical application or research in the abstract).

##### 3.2.2.3. Example for rating level d

“*Furthermore, a high level of satisfaction with the individual’s local infrastructure may support the residents to engage in higher levels of physical activity for transportation, whereas the preferred mode of transportation may be gender-specific: men tend to use the bicycle while women walk. Our results suggest that local infrastructure facilities should be designed so as to ensure accessibility by both walking and cycling*.” (35) (abstract).

### 3.3. Introduction

#### 3.3.1. (4) Rationale for the consideration of sex/gender given (possible rating levels a, b, c)

At the beginning of a publication, authors should state why sex/gender might be of interest for their research (3, 25). This enables the reader to place the publication in its scientific background and to assume how the particular study contributes to the existing body of evidence (25). A rationale for the consideration of sex/gender might be based on previous evidence or theoretical considerations and should be formulated before starting with the analysis. However, due to word limitations researchers might only chose an implicit mention of sex/gender, e.g., under the terms of sociodemographic factors or intersectionality (rating level b). These publications should be distinguished from those that explicitly refer to sex/gender within their rationale (rating level c). If no explanation for the consideration of sex/gender is given, the publication is rated “a: not at all.”

##### 3.3.1.1. Example for rating level b

“*Dietary patterns show specific associations with sociodemographic, lifestyle and other health factors that can help identify subgroups for nutrition guidelines in public health […]*.” (36) (p. 5).

##### 3.3.1.2. Example for rating level c

“*The relevance of depression disorder during pregnancy as well as the link between gender norms and mental health led us to explore this potential association in a specific and vulnerable collective, that is, pregnant women*.” (37) (p. 810).

#### 3.3.2. (5) Sex/gender mentioned in the objectives (possible rating levels a, b, c)

The objectives include a clear and explicit statement of what the authors intend to investigate (38). If they are planning to put a special focus on the analysis of sex/gender, they need to indicate this in the objectives (26). Similar to the rationale, sex/gender might be considered implicitly (rating level b) or explicitly (rating level c). An implicit mention is given when sex/gender is included, for example, under the terms of socio-demographic factors or intersectionality. If the analysis of sex/gender is not stated as one of the study’s objectives, the publication is rated “a: not at all.”

##### 3.3.2.1. Example for rating level b

“*The aim of this paper is to identify protective and non-protective identity intersections and to determine the strength of relationships between social identities and intersections, and lifetime substance use as well as high-risk and harmful alcohol use*.” (39) (p. 623).

##### 3.3.2.2. Example for rating level c

“*The purpose of this study was […] to assess whether the use of green spaces differs among men and women, after adjustment for sociodemographic and health-related variables.”* (40) (p. 670).

#### 3.3.3. (6) Sex/gender considered in formulating the hypotheses (possible rating levels a, b)

An explicit report of sex/gender related assumptions and underlying hypotheses will help the reader understand the choice of methods and interpretation of results (26). In order to achieve rating level b for this criterion, authors need to state the direction of sex/gender-specific findings they are expecting to detect. If sex/gender was not considered at all in the process of formulating the study’s hypotheses, the publication receives the rating “a: not at all.”

In this context, authors need to clarify whether the analyses are conducted exploratory (with the purpose of finding new relationships) or confirmatory (with the purpose of testing one or more hypotheses) (41). In case of an exploratory analysis this criterion is probably not applicable because the formulated objectives and hypotheses are less specific (25). However, regardless of the type of research, we recommend reporting any assumptions that have been made regarding a study’s findings.

##### 3.3.3.1. Example for rating level b

“*We hypothesized that regardless of their living environment and biological sex, older adults endorsing the androgynous gender role will have lower prevalence of depression due to better psychological adaptation and higher competence*.” (42) (p. 15).

### 3.4. Methods

#### 3.4.1. (7) Source of information for recruitment is reported (possible rating levels a, b)

To achieve adequate representation of different sex/gender groups in their study population, researchers might recruit their participants stratified by sex/gender (43). In this case, it is necessary to report the sources of information used to identify study participants (41, 44). To meet rating level b of this criterion authors need to report the source of sex/gender information they used for the recruitment of their study population. If sex/gender was considered in the recruitment process, but no further information was given, the publication has to be rated with rating level “a: not at all.” However, if no sex/gender-specific recruitment was performed, this criterion should be marked as not applicable in the matrix.

##### 3.4.1.1. Example for rating level b

“*The random sample was from a national population register, stratified by gender and age, consisting of 3,254 men and 3,907 women aged 45–72 residing in Kaunas city, Lithuania*.” (40) (p. 670).

#### 3.4.2. (8) Reporting of selection of study population (possible rating levels a, b)

The validity of a study’s findings highly depends on the choice of study participants (44). Hence, readers need information on the decisions made regarding the composition of the study population to assess the findings’ generalizability (25, 41, 44). Depending on the research question and the recruitment strategy authors might not always specifically address sex/gender. Thus, we distinguish between three different cases with corresponding requirements that have to be met to fulfill rating level b:

In the first case, authors aim for representativity by inviting the entire source population or a random sample that they have drawn from it (44). For those studies, we consider rating level b to be fulfilled, if authors especially state the aim of representativity, adequately report their methods of recruitment and assess how well the study sample reflects on the source population from which it was drawn. Here, it is necessary to pay special attention to the sex/gender composition, i.e., authors need to report if the study population’s sex/gender composition matches the distribution in the source population. Information on the population’s representativity might be found in the results section of a publication.

In the second case, all of the considered information is taken from register data without the need to ask the included population for their participation. In this case, a statement on the register’s representativeness is necessary.

In the third case, authors do not aim for representativity. Here, they need to describe the considerations made in regard of the sex/gender distribution of their study population to fulfill the requirements of rating level b. All methods of selecting the study population (e.g., exclusion or over-sampling) need to be justified with regard to the research question (25, 44). A rationale must be given if any sex/gender groups were excluded or treated differently (3, 44, 45).

In case of a longitudinal study, methods of follow-up should be described (25). If a study includes several study populations, recruitment information has to be provided for each of them to consider this criterion fulfilled.

If none of the requirements that have been described above are met the publication is rated with level “a: not at all.”

##### 3.4.2.1. Example for rating level b, case 1

“*As cases, we used deaths from myocardial infarction (ICD-10: I21-I22), hypertension (ICD-10: I10-I15), and Type II diabetes mellitus (ICD-10: E10-E14) which occurred in Barcelona between 2004 and 2007. […] The information was obtained from death certificates collected by the Catalan Mortality Register.”* (46) (p. 195).

##### 3.4.2.2. Example for rating level b, case 2

“*The study, which was conducted in accordance with the Swedish law of ethics, was based on data from an extensive public health survey distributed as a mailed questionnaire in the Scania region in southern Sweden. All individuals 18–80 years old, living in this region on 30 June 2004, constituted the study population (N = 855,599). The population was stratified by gender and geographical area, resulting in 2,662 = 124 different strata. Samples were randomly selected from the population registry such that an approximately equal number of individuals were contacted in each stratum*. […] *The participation rate was higher among women, the elderly, individuals born in Sweden and among individuals with high education and income*.” (34) (p. 1).

##### 3.4.2.3. Example for rating level b, case 3

“*We suspected that participants with an intersex condition or non-conform gender identity are more likely to have discrepancies between their psychosocial and biological sex, thus these were excluded from the analyses to compose the gender index.”* (47) (p. 2).

#### 3.4.3. (9) Data collection and operationalization reported (possible rating levels a, b, c)

The way variables of interest are measured affects the findings’ reliability and validity (25). Especially with regard to sex/gender, the participants’ self-assessment may differ from that of an interviewer or the entry in an official register (48). In order to allow for replication and comparability to other studies, authors need to provide a brief description of the source from which relevant information, in this case on sex/gender, was retrieved or how sex/gender was measured in the study (rating level b) (26, 41, 44, 49). Researchers might have already collected sex/gender information for the recruitment of the study population (see also criterion 7). However, in order to meet the requirements of rating level b, authors need to indicate whether the sex/gender-related information applied in the recruitment process was also used for the analyses or whether additional information was collected.

To avoid confusion and increase comparability authors need to state which dimensions of sex/gender have been assessed and how they have been operationalized, i.e., which questions were asked and which categories were offered for response (3, 26). To meet rating level c, authors need to give a brief description of all sex/gender-related items; a simple referral to the sex/gender distribution is not sufficient.

If the study to be evaluated includes several different study populations, information of data collection and operationalization have to be provided for each of them. However, if both information on data collection and on operationalization are lacking, the publication is rated “a: not at all.”

##### 3.4.3.1. Example for rating level b

“*The information was obtained from death certificates collected by the Catalan Mortality Register. We only considered the death certificates of Barcelona city residents who died in the city between 2004 and 2007. Each certificate included the age, sex, the (last) residential address and the underlying cause of the individual’s death*.” (46) (p. 195).

##### 3.4.3.2. Example for rating level c

“*Participants completed the two-question assessment of gender identity […] to determine their gender self-categorization. The 2QAGI asks participants to indicate both their current gender identity and birth-assigned gender category as separate items. The first item asked: What is your current identity? The response options were female, male, transgender female, transgender male, genderqueer, intersex. The second item asked: What gender category were you assigned at birth? The response options were female, male, and intersex*.” (50) (p. 227).

#### 3.4.4. (10) Consideration of sex/gender variety and multidimensionality (possible rating levels a, b, c)

If authors operationalize sex/gender by using a binary one single item that distinguishes between men and women and do not consider its variety and multidimensionality, this criterion is rated with rating level “a: not at all.” The binary distinction of sex/gender into men and women is criticized as it does not do justice to the lived reality (48). For this reason, in some surveys a third category (e.g., diverse) was introduced (51, 52). We consider level b of this criterion to be fulfilled if authors measure sex/gender with a single item question that distinguishes between male/masculine/man, female/feminine/woman and a third category, such as diverse or other.

However, there might be some exceptions to this criterion. One example is the question about the sex assigned at birth. Even though there have been legal changes in some countries, for a long time, infants were either assigned male or female, when they were born. As a consequence, in many populations this question can only be answered with two different categories (48). We therefore recommend that rating level b of this criterion is considered fulfilled if a binary assessment of sex/gender has been sufficiently justified.

As sex/gender is characterized by a great variability and multidimensionality (1), there is a need to apply more sophisticated measurements to adequately capture them (48). If authors apply more advanced instruments to measure sex/gender variety and multidimensionality than the mere differentiation into the aforementioned three categories, they fulfill the requirements of rating level c.

##### 3.4.4.1. Examples for rating level b

“*In order to describe gender differences, in GEDA 2019/2020-EHIS the gender identity was used. Respondents could indicate the gender with which they identify. Among the respondents 18 years and older, 11,959 were women and 10,687 were men. Sixty-two respondents identified with a different gender or did not provide any information*.” (53) (authors own translation, p. 30).

##### 3.4.4.2. Examples for rating level c

“*We queried respondents’ sex and gender separately using a two-step approach […]. Respondents were asked, “What sex were you assigned at birth? (For example, on your birth certificate).” The possible responses were female, male, and intersex; […]. Respondents were then asked, “What is your current gender?,” with the options woman, man, transgender, and a gender not listed here*.” (54) (p. 105).

#### 3.4.5. (11) Implementation of sex/gender specific analysis (possible rating levels a, b, c)

In order to clearly understand the findings, readers need a brief description of all statistical methods (25). Thus, authors should indicate which methods or approaches they have used with the aim of answering their research questions (6, 20). If sex/gender is considered as confounding variable, we consider rating level b to be fulfilled. However, if a more thorough analysis of sex/gender is required, the methods might go beyond the simple consideration of sex/gender as a confounder (55). To meet the requirements of rating level c, at least one sex/gender related variable needs to be considered as an independent influencing factor, for subgroup or interaction analysis or within a comparable analysis methodology. If no information on sex/gender-specific analysis was given within the publication, it receives the rating “a: not at all.”

##### 3.4.5.1. Example for rating level b

“*The combined impacts of allotment gardening and age on measures of health, well-being and physical activity were estimated in a covariate adjusted general linear model (ANCOVA) with allotment gardening (allotment gardeners/neighbors) and age (<62 yrs/≥ 62 yrs) as factors and gender, education level, income, access to a garden at home, physical activity in winter, and stressful life events as covariates*.” (56) (p. 5).

##### 3.4.5.2. Example for rating level c

“*Since previous studies indicate that the effect of SES on change in PA may differ by gender (…) we conducted all analyses for men and women separately*.” (57) (p. 3).

### 3.5. Results

#### 3.5.1. (12) Description of the study population (possible rating levels a, b)

Readers need details on the study population to judge the findings’ generalizability (25). Thus, in order to fulfill rating level b authors should report the sex/gender distribution by stating the number or proportion of study participants within each sex/gender category (6). If neither the results section nor the added tables and figures contain any information on the sex/gender distribution of the study population, the publication is rated with an “a: not at all.”

##### 3.5.1.1. Example for rating level b

“*All of the 1967 participants (1,025 women and 942 men) were born between 1938 and 1947, and the mean age for men (69.13, 2.92 SD) and women (69.10, 2.80 SD) was approximately similar*” (58) (p. 7).

#### 3.5.2. (13) Sex/gender-specific results presented (possible rating levels a, b, c, d)

Authors should indicate at least one relevant baseline characteristic, such as confounder, exposure, outcome and – if relevant – missing data disaggregated by sex and/or gender (rating level b) (59). This allows the reader to better understand the measures of association and to assess the findings’ generalizability (25).

To achieve rating level c, authors need to report the findings of the sex/gender-specific analysis. Findings should be reported regardless of whether they are positive, negative or “null” findings. However, researchers should choose an adequate and balanced presentation of sex/gender results (3, 6). If, for instance, the analysis of effect modification of sex/gender did not yield any significant results, it might be enough to simply state this with one sentence without further displaying the findings. Since we consider the reporting of baseline characteristics and findings of the sex/gender-specific analysis to be equally important, the two rating levels are regarded as equivalent. If authors give information on both, the distribution of baseline characteristics stratified by sex/gender and findings of the sex/gender-specific analysis, the publication is rated with rating level d. If neither is given, the publication receives rating level “a: not at all.”

##### 3.5.2.1. Example for rating level b

See Babisch et al. (60) (Table 1).

##### 3.5.2.2. Example for rating level c

See de Kluizenaar et al. (61) (Tables 2–4).

**Table 2:** Assessment matrix for the consideration of sex/gender in publications assessing sex/gender in the association between residential green space and self-rated health.

ID	Precise sex/gender terms used	Sex/gender in the title	Sex/gender in the abstract	Sex/gender in the rationale	Sex/gender in the objectives	Sex/gender in the hypotheses	Recruitment information described	Sex/gender- specific recruitment described	Source of sex/gender information reported	Sex/gender dimensions/ variability considered	Sex/gender analysis reported	Sex/gender distribution reported	Sex/gender findings reported	Sex/gender findings discussed
	a b	a b	a b c d	a b c	a b c	a b	a b	a b	a b c	a b c	a b c	a b	a b c d	a b c
1	a	a	c	c	c	a	b	b	a	a	c	b	c	c
2	a	a	c	c	c	a	b	b	a	a	c	b	d	b
3	a	a	b	a	a	a	NA	b	a	a	c	b	d	a
4	a	a	c	a	c	a	b	b	a	a	c	b	d	b
5	a	a	c	c	c	a	NA	b	b	a	c	b	c	b
6	a	a	d	a	a	a	b	b	b	a	c	b	d	c
7	a	a	c	c	c	a	a	b	b	a	c	b	c	c

Assessment matrix for the consideration of sex/gender, *n* = 7. 1: Bjork et al. (34), 2: Dadvand et al. (62), 3: Orban et al. (63), 4: Reklatiene et al. (40), 5: Ruijsbroek et al. (64), 6: Stronegger et al. (35), 7: Triguero-Mas et al. (65); the publications were identified by the systematic review of Bolte et al. (18); fulfillment of the evaluation criterion; a- not at all; b, c, or d – to a certain extent depending on the specific criterion (for detailed explanation see results section).

**Table 3:** Assessment matrix for the consideration of sex/gender in publications assessing sex/gender in the association between environmental noise and cardiovascular disease.

ID	Precise sex/gender terms used	Sex/gender in the title	Sex/gender in the abstract	Sex/gender in the rationale	Sex/gender in the objectives	Sex/gender in the hypotheses	Recruitment information described	Sex/gender- specific recruitment described	Source of sex/gender information reported	Sex/gender dimensions/ variability considered	Sex/gender analysis reported	Sex/gender distribution reported	Sex/gender findings reported	Sex/gender findings discussed
	a b	a b	a b c d	a b c	a b c	a b	a b	a b	a b c	a b c	a b c	a b	a b c d	a b c
1	a	a	a	a	a	a	NA	b	b	a	c	b	b	b
2	a	a	d	b	c	a	NA	a	b	a	c	b	d	c
3	a	a	b	a	a	a	b	b	b	a	c	b	d	b
4	a	a	a	a	c	a	NA	a	a	a	c	b	c	b
5	a	a	a	c	c	a	NA	a	a	a	c	b	c	b
6	a	a	a	a	a	a	NA	a	a	a	c	b	c	b
7	a	a	a	a	a	a	NA	a	a	a	c	b	c	c
8	a	a	a	a	a	NA	a	a	a	a	c	b	d	b
9	a	a	a	a	a	a	a	a	b	a	c	b	c	b
10	a	a	a	a	a	a	NA	a	a	a	c	b	c	b
11	a	a	b	c	a	a	NA	a	a	a	c	b	a	a

Assessment matrix for the consideration of sex/gender, *n* = 11. 1: Babisch et al. (60), 2: Banerjee et al. (66), 3: Barcelo et al. (46), 4: de Kluizenaar et al. (61), 5: Evrard et al. (67), 6: Foraster et al. (68), 7: Jarup et al. (69), 8: Pitchika et al. (70), 9: Pyko et al. (71), 10: Sörensen et al. (72), 11: Zeeb et al. (73); the publications were identified by the systematic review of Rompel et al. (15). Fulfillment of the evaluation criterion; a- not at all; b, c, or d – to a certain extent depending on the specific criterion (for detailed explanation see results section).

##### 3.5.2.3. Example for rating level d

See Stronegger et al. (35) (Tables 1–3).

### 3.6. Discussion

#### 3.6.1. (14) Discussion of sex/gender-specific results (possible rating levels a, b, c)

To fulfill the requirements of rating level b, authors need to summarize the findings of the sex/gender-specific analysis. A short summary of the study’s sex/gender results reminds the reader of the main findings and provides an entry point for the following discussion. To put the findings in a context, it might be useful to also report sex/gender results that were identified in similar studies (25).

To fulfill the requirements of rating level c the authors need to discuss the findings beyond a simple summary of results, i.e., the findings need to be interpreted against the background of evidence from previous studies or theoretical considerations (25). Researchers should reflect on which dimensions of sex/gender might be of relevance for the interpretation of their findings and, if applicable, interpret sex and gender differences by considering biological plausibility and social context (74, 75). In order to avoid reproducing any sex/gender-related stereotypes, authors should be careful with speculations without an empirical foundation (45). It might also be appropriate to discuss the findings’ generalizability and their potential implications for future research or practice (25). Since we consider these levels as increasing in importance, publications should always be rated with the highest level whose requirements they meet. If sex/gender was not considered at all within the discussion section, the publication receives rating level “a: not at all.”

##### 3.6.1.1. Example for rating level b

“*Residential surrounding greenness and subjective residential proximity to green spaces were associated with better SGH. We found indications for mediation of these associations by mental health status, perceived social support, and to less extent by physical activity. These mediators appeared to be more relevant for the impact of residential surrounding greenness than subjective proximity to green spaces. We also observed some indications for variations in these mediation roles across strata of sex and age. We did not observe any association between objective residential proximity to green spaces and SGH*.” (62) (p. 165).

##### 3.6.1.2. Example for rating level c

“*When stratified by gender and degree of urbanization, there was a tendency for slightly stronger associations between green spaces and health for women and those living in non-densely populated areas, but this was not statistical significant. The small differences we found by gender are consistent with previous findings that have suggested that women use green spaces more because they are more likely to take care of older people and children than men […]. However, some other studies have found that green spaces are more used and more beneficial for males […].”* (65) (p. 39).

### 3.7. Exemplary application

Tables 2, 3 display the exemplary application of the assessment matrix to the seven and eleven publications that were included in the systematic reviews by Bolte et al. (18) and Rompel et al. (15), respectively. The different rating levels of each criterion are highlighted by different shades of blue with the darkest shade always being the highest level that could be achieved for this criterion. Level “a: not at all” is always displayed by the lightest shade. As already described, criteria 13: “Sex/gender-specific results presented” is an exception. Since the reporting of baseline characteristics and findings of the sex/gender-specific analysis are seen as being equally important, they are both displayed in the same shade of blue.

**Figure 1:**
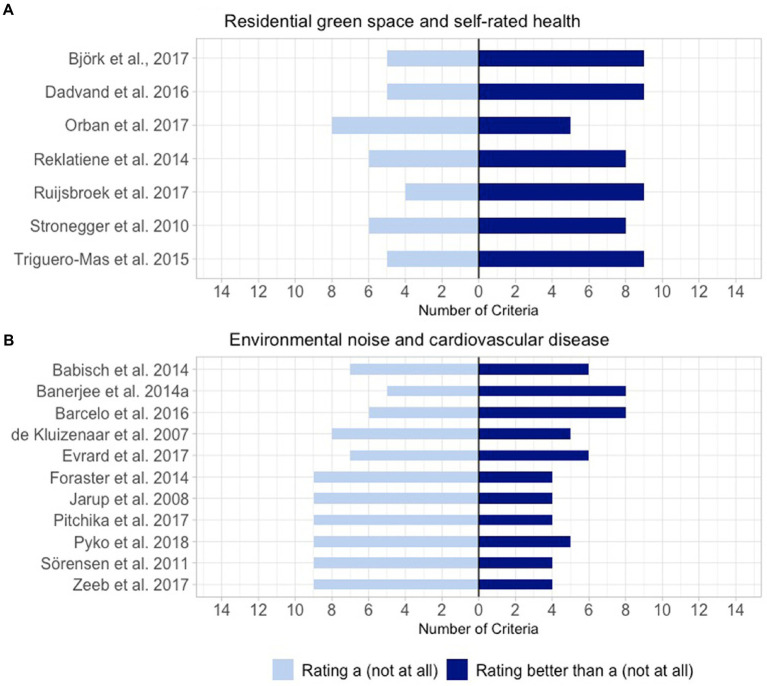
Number of criteria rated as better than “a: not at all” per publication. **(A)** Publications identified by the systematic review of Bolte et al. (18) assessing sex/gender in the association between residential green space and self-rated health (*n* = 7). **(B)** Publications identified by the systematic review of Rompel et al. (15) assessing sex/gender in the association between environmental noise and cardiovascular disease (*n* = 11).

**Figure 2:**
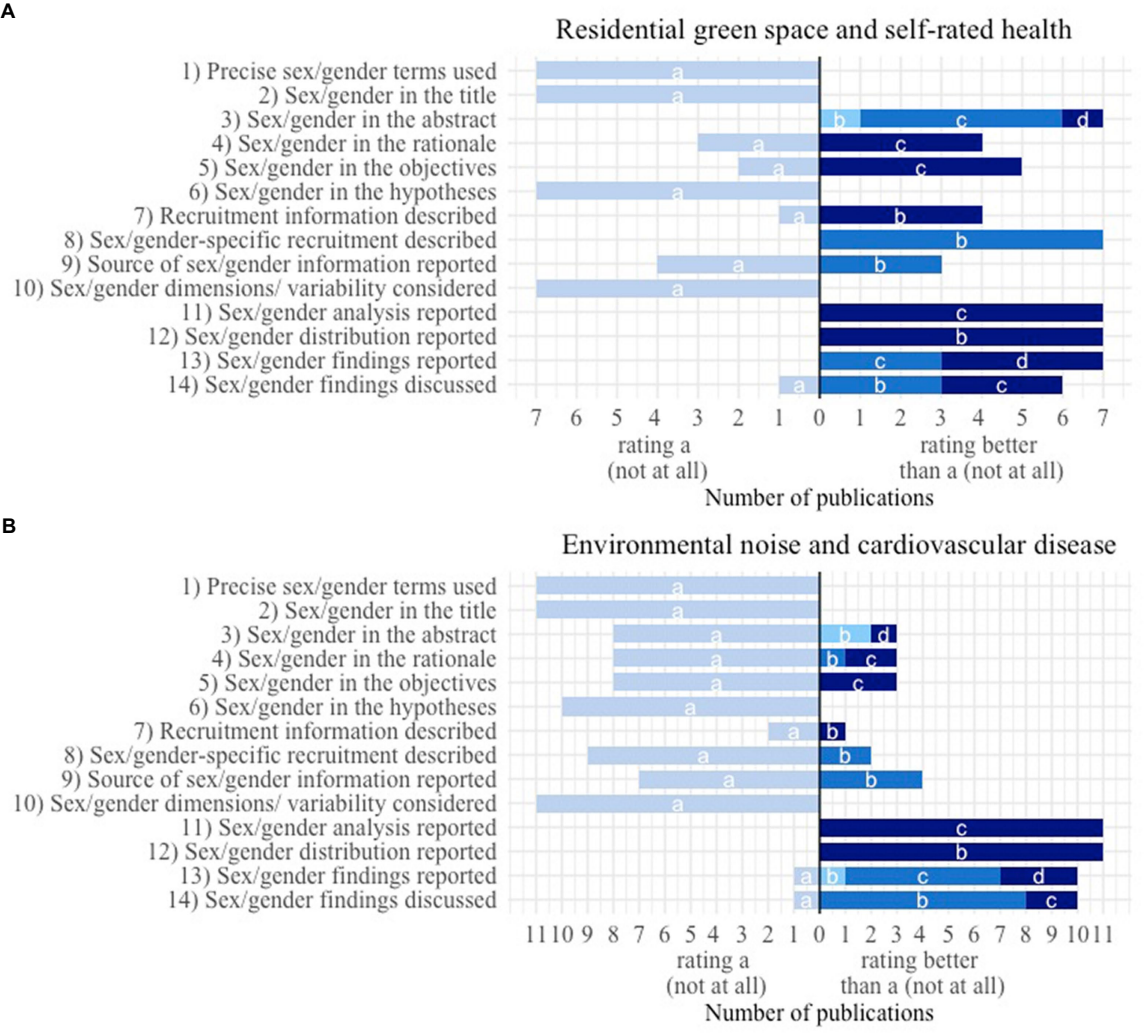
Number of publications with a specific rating of the 14 criteria to assess sex/gender consideration. Fulfillment of the evaluation criterion: a – not at all; b, c or d – to a certain extent depending on the specific criterion (for a detailed explanation see results section). **(A)** Publications identified by the systematic review of Bolte et al. (18) assessing sex/gender in the association between residential green space and self-rated health (*n* = 7). **(B)** Publications identified by the systematic review of Rompel et al. (15) assessing sex/gender in the association between environmental noise and cardiovascular disease (*n* = 11).

Figures 1A,B display the number of criteria rated better than “a: not at all” per publication. Figures 2A,B show how often the requirements of the different rating levels were achieved for each criterion. For both figures we distinguished between rating level “a: not at all” and the other rating levels.

The two exemplary matrices allowed us to compare the consideration of sex/gender within the publications that have been included in the systematic reviews of Bolte et al. (18) (Table 2) and Rompel et al. (15) (Table 3), respectively. The publications from the systematic review of Bolte et al. show better ratings on average which is especially true for the first half of the table. This displays the different eligibility criteria that have been applied for both reviews allowing only publications that mention sex/gender in title or abstract to be included in the systematic review of Bolte et al. while for the review of Rompel et al. it was sufficient if sex/gender-specific effect estimates were calculated in the study. Both tables have in common that none of the included publications achieved a rating better than “a: not at all” for any of the four criteria (1) “Precise use of sex/gender-specific terminology used,” (2) “Sex/gender mentioned in the title,” (6) “Sex/gender considered in formulating the hypotheses” and (10) “Consideration of sex/gender variety and multidimensionality.”

Based on the experiences gained during the development process and by the exemplary application, we have compiled recommendations for the use of the assessment matrix (Box 1).

**BOX 1**

Recommendations for use of the assessment matrix.

- We recommend two reviewers to independently conduct the evaluation and to solve disagreements by discussion. It might be useful to note the text passages that are relevant for the evaluation.
- Sometimes authors might not provide all the information, which users of the matrix will need for their evaluation. In these cases, we recommend rating the according criteria “a: not at all.”
- We recommend considering both the main manuscript and Supplementary material to gather information for the evaluation. In some cases, authors refer to previous publications where further aspects of a study’s methodology have already been described. If these publications are easily accessible, they should also be taken into account in the process of evaluation.
- In order to maintain a good overview and to reveal further patterns users might consider stratifying the publications to be assessed based on further characteristics (e.g., long-term or short-term studies).
- When using the matrix, we recommend to carefully choose the eligibility criteria, to make sure to report them transparently and to discuss the resulting implementations and limitations.

## 4. Discussion

For a standardized, comprehensive evaluation of sex/gender consideration in publications of quantitative health-related research we developed an assessment matrix in an interdisciplinary cooperation. We provided a rationale and an explanation for each criterion of the matrix as well as a tool to visualize the evaluation results including R code. Furthermore, to test the practicability of the matrix, we applied it in an exemplary manner to studies of two systematic reviews in the research field environmental health (15, 18). In recent years, several guidelines have been published that support researchers in including sex and gender more systematically in the entire research process and it’s reporting (3, 5–7). By introducing the matrix as a flexible instrument, we hope to provide assistance to other researchers in evaluating a publication’s adherence to these guidelines, in comparing the consideration of sex/gender within different publications and fields of research, and in identifying areas of study planning and reporting that are already well met and those that require further effort. Furthermore, parts of the assessment matrix might also be applicable for decision makers evaluating grant applications for studies.

During the last years, various different guidelines and checklists were developed to encourage and support researchers in considering sex/gender in their research (3, 5, 13). Moreover, an increasing number of academic journals require the reporting of sex/gender within their editorial policies (9, 76, 77). In response to these developments, there is an increase in publications aiming to assess how these suggestions of considering sex/gender have been taken into account in different fields of research (14, 15, 17–19). However, within these publications the authors mostly assessed sex/gender with a set of criteria, which they had developed especially for this particular publication. None of them made the claim of introducing a set of criteria that is independent of the studies’ objectives and applicable to quantitative studies in different fields of health research.

An evaluation strategy most comparable to our approach was developed by Day et al. (20), who, however, focused on the assessment of sex/gender consideration in research proposals and do not include any research articles. Nevertheless, they introduced a useful set of requirements that a tool for the assessment of sex/gender consideration must meet covering its completeness, comprehensibility and utility. Hereinafter we refer to this set of requirements to discuss our newly developed assessment matrix against the background of hitherto existing evaluation instruments.

### 4.1. Completeness

Current guidelines are encouraging researchers to consider sex/gender throughout every step of conducting and reporting a study (5, 10, 11). Consequently, a tool applied to evaluate sex/gender consideration needs to capture each step of the research process (20). We found that most publications describing the evaluation of sex/gender consideration already cover several steps of the research process (15, 17, 18). However, we noticed that they quite often focus on special aspects of sex/gender consideration such as the adequate and precise use of terminology (24).

To ensure that all research steps are adequately taken into account, we followed the STROBE guidelines to structure our matrix into the sections title, abstract, introduction, methods, results and discussion (25). For every section we developed corresponding criteria based on the expertise represented in our interdisciplinary research team and currently published guidelines concerning the good reporting of health-related studies (25) or the adequate consideration of sex/gender (3, 5).

However, we see the matrix as an evolving instrument. The awareness of the importance of sex/gender for health-related research is steadily increasing and health-related sex/gender research is in a state of constant change and further development (2, 78, 79). In addition, some fields of research and study objectives might require additional criteria for assessment in the matrix. Hence, we strongly recommend researchers using our matrix to review the requirements of the particular research area in which they are interested and to refine or complement the assessment criteria if necessary. Since the criteria that are used to review a manuscript have an influence on the resulting findings, we recommend that authors choose them carefully and discuss the consequences of this choice.

A possible addition to our matrix might be the consideration of other social categories in the sense of intersectionality (80). The interconnectedness of sex/gender with other social categories and power relations is considered important in exploring the pathways leading to different health related outcomes (4, 14). Hence, it is not recommended to consider sex and gender as operating in isolation but as intersecting with other social determinants (80). One example for the consideration of intersectionality can be found in the set of criteria that Williams and colleagues (19) established to evaluate the use of sex and gender in health policymaking. Here they ask whether and which other axes of intersectionality besides sex/gender have been taken into account.

### 4.2. Comprehensibility

Day and others stress out that a tool for the assessment of sex/gender needs to be easy to use and understand (20). Merriman and colleagues conducted a review of sex/gender reporting and author representation in leading general medical and global health journals. Here, they noticed the difficulties to quantify and standardize the performance of sex/gender analysis (14). With our matrix we faced similar issues. In order to allow for different gradations in the assessment, we included different levels for several of our matrix’ criteria. A similar approach can be found in the “Essential Metrics for Assessing Sex & Gender Integration in Health Research Proposals Involving Human Participants” that was developed by Day et al. (20). They used a scale ranging from the four categories “poor,” “fair,” “good” to “excellent” to rate their criteria. Williams et al. (19) applied a Likert scale from one to five to assess the precise use of sex/gender terminology. However, for some publications and criteria, the order of assessment levels might be inconclusive. In the example of criteria 4 and 5, an implicit mention of sex/gender that is embedded in the context of intersectionality might be more appropriate than an explicit mention of sex/gender related keywords without a theoretical basis. We recommend that users of the matrix highlight these cases if they occur and discuss the resulting implications.

Since we aimed to construct a set of assessment criteria that can be applied without the necessity of deeper sex/gender theoretical knowledge, the matrix does not provide deeper analysis of the consideration of sex/gender. Despite, for example, assessing the use of instruments that measure sex/gender variety and multidimensionality, the matrix does not include the evaluation of these instrument’s appropriateness. Thus, even though authors might consider different dimensions of sex/gender, and their publication therefore receives the highest possible rating, the applied instruments might still not be the best fit for the particular research question. Similarly, the highest rating for the criterion covering the section of the findings’ discussion does not give any indication of its adequacy and completeness nor does it consider if any implications of the studies’ results were described.

Users aiming for a deeper qualitative evaluation of sex/gender consideration might therefore consider supplementing the matrix with further analysis. Nevertheless, we consider the matrix to be a useful starting point to identify issues that need more focus.

### 4.3. Utility

Day et al. (20) stress out the requirement for a tool developed for the assessment of sex/gender consideration to be useful for researchers aiming to adequately consider sex/gender within their own research as well as for reviewers who aim to evaluate other publications. We especially consider the graphical visualization of the evaluation of sex/gender consideration as essential for the matrix’ usability since graphics can be used to identify overall patterns and outlying observations (21, 22). Moreover, graphics can be tailored specifically to different audiences and their respective needs (81). Some of the other authors assessing the consideration of sex/gender already used bar plots to display single aspects of their evaluation (17, 19). However, to our knowledge our publication is the first that introduces a graphical systematic to display the evaluation of sex/gender consideration within one graphic.

Nevertheless, the matrix might get confusing when too many publications are included, especially when exceeding the size of one page. To address this issue, we introduced two additional graphics that are indented to summarize the results from different angles. Firstly, with the aim of getting an impression of a publication’s overall performance we displayed how many times they achieved a rating better than “a: not at all.” This way weaker and stronger publications in the sense of sex/gender consideration can be identified. Nonetheless, one has to keep in mind that within this way of presentation all criteria are treated as equally important. Researchers might, however, consider weighting certain criteria as more important than others depending on their particular field of study. The second additional graphic illustrates how each criterion is evaluated in the specific set of publications. This might help to provide a better overview about overall strengths and weaknesses of the included publications in the light of the consideration of sex/gender.

Similar to systematic reviews the matrix is based on the manual assessment of the included publications (82). Hence, the number of articles that could be included is limited to the resources within a research project (14). Therefore, we recommend to carefully consider the eligibility criteria chosen to select the publications to be assessed and included in the matrix.

In this context, it must also be considered that the inclusion criteria might influence the matrix’ findings. For both of the reviews that we included for the exemplary application of the assessment matrix different eligibility criteria were applied. In the review by Bolte et al. (18) that analyses sex/gender in the association between green spaces and self-rated health publications needed to mention at least one keyword for sex/gender in the title or abstract which was not necessary for the review about environmental noise exposure on hypertension and ischemic heart disease by Rompel et al. (15). Consequently, the assessment of both sets of publications differ especially with regard to the criteria assessing the consideration of sex/gender in the abstract, where on average the publications on green spaces and self-rated health show better ratings. This shows how a different focus of study selection might influence the assessment matrix’ appearance. Nevertheless, both reviews have in common that they only included publications calculating sex/gender-specific effect estimates. All studies that solely considered sex/gender as confounding variable were excluded. Since the reviews’ eligibility criteria already overlap with the criteria of the assessment matrix no conclusions about analysis methods can be made on the basis of our examples. Consequently, we recommend carefully choosing the eligibility criteria, making sure to report them transparently, and discussing the resulting limitations.

With the exemplary application of the matrix to publications dealing with sex/gender in the association between residential green space and self-rated health and between environmental noise and cardiovascular disease, respectively, we found that none of the publications did consider the variety and multidimensionality of sex/gender. This result shows that existing operationalization possibilities are limited and that improvements are needed in this area. Accordingly, current developments show that scientists in quantitative health research increasingly consider several dimensions of gender and that corresponding tools are being developed (76). For instance, the research project INGER addresses this research gap by developing a multidimensional sex/gender concept for the operationalization of sex and gender in quantitative (environmental) health research (1) as a basis for defining a set of variables and for applying different analytical methods for their analyses (83).

## 5. Conclusion

We developed an assessment matrix for the systematic and comprehensive evaluation of sex/gender consideration in quantitative health-related studies. By introducing the matrix, we aim to provide users with a tool to compare sex/gender consideration between different publications and even different fields of study. In this way, the assessment matrix constitutes a tool to identify research gaps and a basis for future research. By introducing the assessment matrix, we aim to contribute to more sex/gender equitable health-related research. In the future a more systematic integration of sex/gender in research and education is needed. We believe the matrix to be a useful tool to support this process by highlighting strengths and weaknesses of sex/gender consideration in current health-related research and thus identifying issues that require more attention.

## Data availability statement

The raw data supporting the conclusions of this article will be made available by the authors, without undue reservation.

## Author contributions

SH, CH, UK, KP, KJ, LD, AS, and GB: conceptualization and writing—review and editing. SH and UK: data curation, formal analysis, and writing—original draft. GB: supervision. GB, KP, and AS: funding acquisition. All authors contributed to the article and approved the submitted version.

## Funding

The work was supported by the collaborative research project “INGER–Integrating gender into environmental health research” (https://www.uni-bremen.de/en/inger/, accessed on 16 March 2023) was funded by the German Federal Ministry of Education and Research (funding number: 01GL1713).

## Conflict of interest

The authors declare that the research was conducted in the absence of any commercial or financial relationships that could be construed as a potential conflict of interest.

## Publisher’s note

All claims expressed in this article are solely those of the authors and do not necessarily represent those of their affiliated organizations, or those of the publisher, the editors and the reviewers. Any product that may be evaluated in this article, or claim that may be made by its manufacturer, is not guaranteed or endorsed by the publisher.

## Footnotes

^1^ https://www.uni-bremen.de/en/inger
