# Supplementary material for: Consideration of sex/gender in publications of quantitative health-related research: Development and application of an assessment matrix
Source: Front Public Health. 2023 Apr 4;11:992557. doi: 10.3389/fpubh.2023.992557 (PMC10110874; doi:10.3389/fpubh.2023.992557)
Supplement: Supplementary file 1 [file Data_Sheet_1.pdf]

Supplementary Material

Consideration of sex/gender in publications of quantitative health-related research: Development and application of an assessment matrix

Sophie Horstmann, Christina Hartig, Ute Kraus, Kerstin Palm, Katharina Jacke, Lisa Dandolo, Alexandra Schneider, Gabriele Bolte

The assessment matrix is a tool for the visual evaluation of the consideration of sex/gender in health-related quantitative studies.

This document contains detailed instructions on how to visualize the conducted assessment in R by using the package *flextable*. Additionally, it is explained how to create the two additional plots that were introduced in the main manuscript. The first plot displays how often publications have been rated better than "a: not at all" while the second plot shows how often the requirements of the different rating levels have been achieved for every criterion. Please keep in mind that it might be necessary to adjust the syntax if you decide to make changes to the matrix and its criteria.

1.1.1.1 Content

1. Preparations of the data set and loading of required packages
2. Assessment matrix
3. Plot 1: Frequency of criteria that have been rated better than "a: not at all" by publication
4. Plot 2: Frequency of how often the different rating levels have been achieved for every criterion

To apply the introduced syntax, a *csv.document* containing information on the assessment of the included publications is needed. Please see the main manuscript for detailed information on the assessment criteria. Table 1 displays the format in which the document needs to be saved.

Supplementary Table 1: Format of the csv.document

ID	Publication	C_1	C_2	C_3	C_4	C_5	C_6	C_7	C_8	C_9	C_10	C_11	C_12	C_13	C_14
1	author + date														
2	author + date														
3	author + date														

…

The final matrix is printed as a *docx.document*. The two plots are saved as a *PNG* file.

1) Preparations

Clear workspace

```
rm(list = ls())
```

Set up your working directory

This directory must contain the prepared *csv.document*. After creating the matrix and the two additional plots they will be saved within this directory.

```
setwd("…")
```

Create your data set ‘matrix’ by loading the *csv.document*

Import the document containing the information on the publications’ assessment into the data frame ‘matrix’. If the parameter header is set to “TRUE”, then the first row will be treated as the row names. Please check if the import has been successful. The data frame must contain 16 columns and as many rows as publications included in the assessment process.

```
matrix <- read.csv2("….csv", header = TRUE)
```

Recode missing data as ‘not applicable’ (NA)

It is possible that some of the criteria are not applicable for every publication. In this case they have to be rated as ‘not applicable’. Within the matrix this is displayed by the abbreviation “NA”.

```
matrix[is.na(matrix)] <- "NA"
```

Install and load required libraries

Please find a list of required packages below. The packages are sorted according to the section of this syntax they are needed for.

- Assessment matrix
  - flextable (Creating tables)
- Plot 1 and Plot 2
  - tidyr (changes format of data set)
  - ggplot (creates plots)
  - dplyr (manipulation of data sets)
  - stringr (manipulation of strings)

```
#install.packages('flextable')
#install.packages('tidyr')
#install.packages('ggplot2')
#install.packages('dplyr')
#install.packages('stringr')

library(flextable)
```

```
library (tidyr)
library(ggplot2)
library (dplyr)
library(stringr)
```

Define the layout for the plots

```
theme_set(theme_minimal(base_family = "", base_size = 12))
```

1.1.2 2) Creation of the assessment matrix

1.1.3 2.1) Create basic flextable (ft) and define the layout

Create the basic flextable (ft)

By using the function *flextable* the table is formatted from the data frame ‘matrix’. The table contains the publications’ ID number and the 14 assessment criteria.

```
ft <- flextable(matrix,
        col_keys = c("ID", "C_1", "C_2", "C_3", "C_4", "C_5", "C_6", "C_7", "C_8", "C_9", "C_10", "C_11", “C_12”
                    ,"C_13", "C_14"))
```

Supplementary table 2: Basic flextable for the first five assessment criteria

ID	C_1	C_2	C_3	C_4	C_5
1	a	a	c	c	c
2	a	a	c	c	c
3	a	a	b	a	a
4	a	a	c	a	c
5	a	a	c	c	c
6	a	a	d	a	a
7	a	a	c	c	c

Define the default layout functions

In a next step the appearance of the table is changed. Feel free to tailor the layout specifically to your audiences and their respective needs.

```
border_header <- fp_border_default(color = "gray41", width = 1.5)
border_body <- fp_border_default(color = "white", width = 0.5)

theme_design <- function(x) {

 #border
 x <- border_remove(x)
 x <- border_inner(x, border = border_body, part = "body")
 x <- hline_bottom(x, part="header", border = border_header )
 x <- hline_top(x, part="header", border = border_header )
```

```
#font
x <- fontsize(x,size = 8, part="all")
x <- font(x, fontname = "Calibri", part = "all")

#position of text in cells
x <- align(x, align = "center")
x <- align(x, j = 1, align = "center")
x <- align(x, align = "left", part = "header")
x <- align(x, align = "center", part = "body")
x <- align(x, align = "left", part = "footer")
x <- set_table_properties(x, layout = "fixed")

#table size
x <- width(x, j = 1, width = 1, unit= "cm")
x <- width(x, j = 2:15, width = 1.75, unit= "cm")
x
}

ft <- theme_design(ft)
```

Supplementary table 3: flextable with new layout

ID	C_1	C_2	C_3	C_4	C_5
1	a	a	c	c	c
2	a	a	c	c	c
3	a	a	b	a	a
4	a	a	c	a	c
5	a	a	c	c	c
6	a	a	d	a	a
7	a	a	c	c	c

1.1.4 2.2) Conditional formatting

The function *bg* is used to change the background color of cells based on a condition. Here, the color of the cells changes based on the achieved rating level. The different rating levels of each criteria are highlighted by different shades of blue (#BCD2EE, #87CEFA, #1874CD, #000080) with the darkest shade (#000080) always being the highest level that could be achieved for this criterion. Level ‘a: not at all’ is always displayed by the lightest shade (#BCD2EE).

```
#Terminology
ft <- bg(ft, ~ C_1 == "a", ~ C_1, bg = "#BCD2EE")
ft <- bg(ft, ~ C_1 == "b", ~ C_1, bg = "#000080")
ft <- color(ft, ~ C_1 == "b", ~ C_1, color = "white")

#Title
ft <- bg(ft, ~ C_2 == "a", ~ C_2, bg = "#BCD2EE")
ft <- bg(ft, ~ C_2 == "b", ~ C_2, bg = "#000080")
ft <- color(ft, ~ C_2 == "b", ~ C_2, color = "white")
```

```
#Abstract
ft <- bg(ft, ~ C_3 == "a", ~ C_3, bg = "#BCD2EE")
ft <- bg(ft, ~ C_3 == "b", ~ C_3, bg = "#87CEFA")
ft <- bg(ft, ~ C_3 == "c", ~ C_3, bg = "#1874CD")
ft <- bg(ft, ~ C_3 == "d", ~ C_3, bg = "#000080")
ft <- color(ft, ~ C_3 == "d", ~ C_3, color = "white")

#Rationale
ft <- bg(ft, ~ C_4 == "a", ~ C_4, bg = "#BCD2EE")
ft <- bg(ft, ~ C_4 == "b", ~ C_4, bg = "#1874CD")
ft <- bg(ft, ~ C_4 == "c", ~ C_4, bg = "#000080")
ft <- color(ft, ~ C_4 == "c", ~ C_4, color = "white")

#Objective
ft <- bg(ft, ~ C_5 == "a", ~ C_5, bg = "#BCD2EE")
ft <- bg(ft, ~ C_5 == "b", ~ C_5, bg = "#1874CD")
ft <- bg(ft, ~ C_5 == "c", ~ C_5, bg = "#000080")
ft <- color(ft, ~ C_5 == "c", ~ C_5, color = "white")

#Hypotheses
ft <- bg(ft, ~ C_6 == "a", ~ C_6, bg = "#BCD2EE")
ft <- bg(ft, ~ C_6 == "b", ~ C_6, bg = "#000080")
ft <- color(ft, ~ C_6 == "c", ~ C_6, color = "white")

#Source Recruitment
ft <- bg(ft, ~ C_7 == "a", ~ C_7, bg = "#BCD2EE")
ft <- bg(ft, ~ C_7 == "b", ~ C_7, bg = "#000080")
ft <- bg(ft, ~ C_7 == "NA", ~ C_7, bg = "white")
ft <- color(ft, ~ C_7 == "b", ~ C_7, color = "white")

#Selection
ft <- bg(ft, ~ C_8 == "a", ~ C_8, bg = "#BCD2EE")
ft <- bg(ft, ~ C_8 == "b", ~ C_8, bg = "#1874CD")
ft <- bg(ft, ~ C_8 == "c", ~ C_8, bg = "#000080")
ft <- color(ft, ~ C_8 == "c", ~ C_8, color = "white")

#Source
ft <- bg(ft, ~ C_9 == "a", ~ C_9, bg = "#BCD2EE")
ft <- bg(ft, ~ C_9 == "b", ~ C_9, bg = "#1874CD")
ft <- bg(ft, ~ C_9 == "c", ~ C_9, bg = "#000080")
ft <- color(ft, ~ C_9 == "c", ~ C_9, color = "white")

#Operationalisation
ft <- bg(ft, ~ C_10 == "a", ~ C_10, bg = "#BCD2EE")
ft <- bg(ft, ~ C_10 == "b", ~ C_10, bg = "#1874CD")
ft <- bg(ft, ~ C_10 == "c", ~ C_10, bg = "#000080")
ft <- color(ft, ~ C_10 == "c", ~ C_10, color = "white")

#Analysis
ft <- bg(ft, ~ C_11 == "a", ~ C_11, bg = "#BCD2EE")
ft <- bg(ft, ~ C_11 == "b", ~ C_11, bg = "#1874CD")
ft <- bg(ft, ~ C_11 == "c", ~ C_11, bg = "#000080")
ft <- color(ft, ~ C_11 == "c", ~ C_11, color = "white")

#Population
ft <- bg(ft, ~ C_12 == "a", ~ C_12, bg = "#BCD2EE")
ft <- bg(ft, ~ C_12 == "b", ~ C_12, bg = "#000080")
```

```
ft <- color(ft, ~ C_12 == "b", ~ C_12, color = "white")

#Results
ft <- bg(ft, ~ C_13 == "a", ~ C_13, bg = "#BCD2EE")
ft <- bg(ft, ~ C_13 == "b", ~ C_13, bg = "1874CD")
ft <- bg(ft, ~ C_13 == "c", ~ C_13, bg = "#1874CD")
ft <- bg(ft, ~ C_13 == "d", ~ C_13, bg = "#000080")
ft <- color(ft, ~ C_13 == "d", ~ C_13, color = "white")

#Discussion
ft <- bg(ft, ~ C_14 == "a", ~ C_14, bg = "#BCD2EE")
ft <- bg(ft, ~ C_14 == "b", ~ C_14, bg = "#1874CD")
ft <- bg(ft, ~ C_14 == "c", ~ C_14, bg = "#000080")
ft <- color(ft, ~ C_14 == "c", ~ C_14, color = "white")
```

Supplementary table 4: flextable with conditional formatting

ID	C_1	C_2	C_3	C_4	C_5
1	a	a	c	c	c
2	a	a	c	c	c
3	a	a	b	a	a
4	a	a	c	a	c
5	a	a	c	c	c
6	a	a	d	a	a
7	a	a	c	c	c

1.1.5 2.3) header and footer

Set labels for the header of the matrix

```
ft <- set_header_labels( ft, C_1 = "Precise sex/gender terms used",
                              C_2 = "Sex/gender in the title",
                              C_3 = "Sex/gender in the abstract",
                              C_4 = "Sex/gender in the rationale",
                              C_5 = "Sex/gender in the objectives",
                              C_6 = "Sex/gender in the hypotheses",
                              C_7 = "Recruitment information described",
                              C_8 = "Sex/gender- specific recruitment described",
                              C_9 = "Source of sex/gender information reported",
                              C_10 = "Sex/gender dimensions/ variability considered",
                              C_11 = "Sex/gender analysis reported",
                              C_12 = "Sex/gender distribution reported",
                              C_13 = "Sex/gender findings reported",
                              C_14 = "Sex/gender findings discussed")
```

Supplementary table 5:flextable with header labels

ID	Precise sex/gender terms used	Sex/gender in the title	Sex/gender in the abstract	Sex/gender in the rationale	Sex/gender in the objectives
1	a	a	c	c	c
2	a	a	c	c	c
3	a	a	b	a	a
4	a	a	c	a	c
5	a	a	c	c	c
6	a	a	d	a	a
7	a	a	c	c	c

Add an additional header row that shows the possible rating levels per criterium

Not every criterion consists of the same amount of rating levels. To avoid confusing we added a header row that contains information on the possible rating levels that could be achieved within the different criteria.

```
ft <- add_header_row(ft, top = FALSE, value = rep("", 15))
ft <- align(ft, align = "center", part = "header", i = 2)

#Terminology
ft <- compose(ft, j =2, i = 2, part = "header",
        value =  as_paragraph(as_highlight(" a ", color = "#BCD2EE"),
                   as_highlight(" b ", color = "#000080")))

#Title
ft <- compose(ft, j =3, i = 2, part = "header",
        value =  as_paragraph(as_highlight(" a ", color = "#BCD2EE"),
                   as_highlight(" b ", color = "#000080")))

#Abstract
ft <- compose(ft, j =4, i = 2, part = "header",
        value =  as_paragraph(as_highlight(" a ", color = "#BCD2EE"),
                   as_highlight(" b ", color = "#87CEFA"),
                   as_highlight(" c ", color = "#1874CD"),
                   as_highlight(" d ", color = "#000080")))

#Rationale
ft <- compose(ft, j =5, i = 2, part = "header",
        value =  as_paragraph(as_highlight(" a ", color = "#BCD2EE"),
                   as_highlight(" b ", color = "#1874CD"),
                   as_highlight(" c ", color = "#000080")))

#Objective
ft <- compose(ft, j =6, i = 2, part = "header",
        value =  as_paragraph(as_highlight(" a ", color = "#BCD2EE"),
                   as_highlight(" b ", color = "#1874CD"),
```

```
                    as_highlight(" c ", color = "#000080")))

#Hypotheses
ft <- compose(ft, j =7, i = 2, part = "header",
        value =  as_paragraph(as_highlight(" a ", color = "#BCD2EE"),
                    as_highlight(" b ", color = "#000080")))

#Source Recruitment
ft <- compose(ft, j =8, i = 2, part = "header",
        value =  as_paragraph(as_highlight(" a ", color = "#BCD2EE"),
                    as_highlight(" b ", color = "#000080")))


#Selection
ft <- compose(ft, j =9, i = 2, part = "header",
        value =  as_paragraph(as_highlight(" a ", color = "#BCD2EE"),
                    as_highlight(" b ", color = "#1874CD"),
                    as_highlight(" c ", color = "#000080")))

#Source
ft <- compose(ft, j =10, i = 2, part = "header",
        value =  as_paragraph(as_highlight(" a ", color = "#BCD2EE"),
                    as_highlight(" b ", color = "#1874CD"),
                    as_highlight(" c ", color = "#000080")))

#Operationalisation
ft <- compose(ft, j =11, i = 2, part = "header",
        value =  as_paragraph(as_highlight(" a ", color = "#BCD2EE"),
                    as_highlight(" b ", color = "#1874CD"),
                    as_highlight(" c ", color = "#000080")))

#Analysis
ft <- compose(ft, j =12, i = 2, part = "header",
        value =  as_paragraph(as_highlight(" a ", color = "#BCD2EE"),
                    as_highlight(" b ", color = "#1874CD"),
                    as_highlight(" c ", color = "#000080")))

#Population
ft <- compose(ft, j =13, i = 2, part = "header",
        value =  as_paragraph(as_highlight(" a ", color = "#BCD2EE"),
                    as_highlight(" b ", color = "#000080")))

#Results
ft <- compose(ft, j =14, i = 2, part = "header",
        value =  as_paragraph(as_highlight(" a ", color = "#BCD2EE"),
                    as_highlight(" b ", color = "#1874CD"),
                    as_highlight(" c ", color = "#1874CD"),
                    as_highlight(" d ", color = "#000080")))

#Discussion
ft <- compose(ft, j =15, i = 2, part = "header",
        value =  as_paragraph(as_highlight(" a ", color = "#BCD2EE"),
                    as_highlight(" b ", color = "#1874CD"),
                    as_highlight(" c ", color = "#000080")))
```

Supplementary table 6: flextable with additional header row

ID	Precise sex/gender terms used	Sex/gender in the title	Sex/gender in the abstract	Sex/gender in the rationale	Sex/gender in the objectives
	a b	a b	a b c d	a b c	a b c
1	a	a	c	c	c
2	a	a	c	c	c
3	a	a	b	a	a
4	a	a	c	a	c
5	a	a	c	c	c
6	a	a	d	a	a
7	a	a	c	c	c

Set a footer

You might choose a footer that contains additional information to the matrix. Here, we added the references that were considered in the matrix.

```
ft <- add_footer_lines(ft,
"Assessment matrix for the consideration of sex/gender, n = 7
1: Björk et al., 2017, 2: Dadvand et al. 2016, 3: Orban et al. 2017, 4: Reklatiene et al. 2014, 5: Ruijsbroek et al. 2017, 6: S
tronegger et al. 2010, 7: Triguero-Mas et al. 2015")

# define layout options for the footer
ft <- color(ft, part = "footer", color = "#666666")
ft <- hline_top(ft, part="footer", border = border_header)
ft <- fontsize(ft,size = 8, part="footer")
ft <- font(ft, fontname = "Calibri", part = "footer")
```

Supplementary table 7: final flextable with footer

ID	Precise sex/gender terms used	Sex/gender in the title	Sex/gender in the abstract	Sex/gender in the rationale	Sex/gender in the objectives
	a b	a b	a b c d	a b c	a b c
1	a	a	c	c	c
2	a	a	c	c	c
3	a	a	b	a	a
4	a	a	c	a	c
5	a	a	c	c	c
6	a	a	d	a	a

ID	Precise sex/gender terms used	Sex/gender in the title	Sex/gender in the abstract	Sex/gender in the rationale	Sex/gender in the objectives
	a b	a b	a b c d	a b c	a b c
7	a	a	c	c	c

Assessment matrix for the consideration of sex/gender applied to these 7 publications of studies: 1 Björk et al., 2017, 2 Dadvand et al. 2016, 3 Orban et al. 2017, 4 Reklaitiene et al. 2014, 5 Ruijsbroek et al. 2017, 6 Stronegger et al. 2010, 7 Triguero-Mas et al. 2015; (63); the publications were identified by the systematic review of Bolte et al (18); fulfilment of the evaluation criterion: a - not at all; b, c or d - to a certain extent depending on the specific criterion (for a detailed explanation see results section).

Write a docx file containing the table

The document is saved in the direction defined at the beginning. It might be necessary to adjust the layout options of your word document.

```
save_as_docx(ft, path = "assessment_matrix.docx")
```

1.1.6 3) Plot 1: Frequency of criteria that have been rated better than ‘a: not at all’ by publication

In this section we describe how to create the bidirectional bar chart displaying the frequency of criteria that have been rated better than “a: not at all” by publication. It requires the data set “matrix” that has been imported in section 1). To successfully apply the following syntax please load the packages *tydr*, *dplyr*, *ggplot* and *stringr*.

1.1.7 3.1) Transformation of the data set

The data set ‘matrix_1a’ is based on the original data set ‘matrix’. It contains the four new variables ‘rating_a’, ‘rating_NA’, ‘total_ratings’ and ‘ratings_better_a’.

New variable: ‘rating_a’: Number of ratings ‘a’ per publication

```
matrix_1a <- matrix %>%
  mutate(rating_a = str_count(C_1, "a") +
                    str_count(C_2, "a") +
                    str_count(C_3, "a") +
                    str_count(C_4, "a") +
                    str_count(C_5, "a") +
                    str_count(C_6, "a") +
                    str_count(C_7, "a") +
                    str_count(C_8, "a") +
                    str_count(C_9, "a") +
                    str_count(C_10, "a") +
                    str_count(C_11, "a") +
                    str_count(C_12, "a") +
                    str_count(C_13, "a") +
                    str_count(C_14, "a"))
```

New variable: ‘rating_NA’: Number of ratings ‘not applicable’ per publication

```
matrix_1a <- matrix_1a %>%
  mutate(rating_NA =  str_count(C_1, "NA") +
```

```
                       str_count(C_2, "NA") +
                       str_count(C_3, "NA") +
                       str_count(C_4, "NA") +
                       str_count(C_5, "NA") +
                       str_count(C_6, "NA") +
                       str_count(C_7, "NA") +
                       str_count(C_8, "NA") +
                       str_count(C_9, "NA") +
                       str_count(C_10, "NA") +
                       str_count(C_11, "NA") +
                       str_count(C_12, "NA") +
                       str_count(C_13, "NA") +
                       str_count(C_14, "NA"))
```

New variable: 'total_ratings': total number of rated criteria per publication

```
matrix_1a$total_ratings = 14- matrix_1a$rating_NA
```

New variable: 'ratings_better_a': Number of ratings better than 'a' per publication

```
matrix_1a$ratings_better_a = matrix_1a$total_ratings - matrix_1a$rating_a
```

Creation of dataset matrix_2a

The data set "matrix_2a" is based on the data set "matrix_1a" and contains the variables 'ID', 'Publication', 'rating_a' and 'ratings_better_a'.

```
matrix_2a <- data.frame("ID" = matrix_1a$ID,
                        "Publication" = matrix_1a$Publication,
                        "rating_a" = matrix_1a$rating_a,
                        "ratings_better_a" =matrix_1a$ratings_better_a)
```

Transformation of dataframe matrix_2a => matrix_3a

The data set 'matrix_3a' is based on the data set 'matrix_2a'. The function *gather* is used to change the form of a data set. Columns are transformed to rows. The column 'rating' now separates between ratings 'a' and ratings better than 'a'. The column 'count' gives information on how often these ratings have been achieved per publication.

```
matrix_3a <- gather(matrix_2a, key="rating", value="count", 3:4)

##    ID   Publication               rating             count
## 1   1   Björk et al., 2017        rating_a           5
## 2   2   Dadvand et al. 2016       rating_a           5
## 3   3   Orban et al. 2017         rating_a           8
## 4   4   Reklatiene et al. 2014    rating_a           6
## 5   5   Ruijsbroek et al. 2017    rating_a           4
## 6   6   Stronegger et al. 2010    rating_a           6
## 7   7   Triguero-Mas et al. 2015  rating_a           5
## 8   1   Björk et al., 2017        ratings_better_a   9
## 9   2   Dadvand et al. 2016       ratings_better_a   9
## 10  3   Orban et al. 2017         ratings_better_a   5
## 11  4   Reklatiene et al. 2014    ratings_better_a   8
## 12  5   Ruijsbroek et al. 2017    ratings_better_a   9
## 13  6   Stronegger et al. 2010    ratings_better_a   8
## 14  7   Triguero-Mas et al. 2015  ratings_better_a   9
```

Preparation and sorting of the variables

For the next step the variable ‘count’ is transformed as numeric and the variable ‘rating’ as factor variable. You might consider sorting the publications. This sorting is also kept in the graphic.

```
matrix_3a$count<-as.numeric(matrix_3a$count)
matrix_3a$rating<-as.factor(matrix_3a$rating)
matrix_3a$Publication <- factor(matrix_3a$Publication, ordered = TRUE,
                                levels = c("Triguero-Mas et al. 2015",
                                           "Stronegger et al. 2010",
                                           "Ruijsbroek et al. 2017",
                                           "Reklatiene et al. 2014",
                                           "Orban et al. 2017",
                                           "Dadvand et al. 2016",
                                           "Björk et al., 2017"))
```

Split data set

We choose a bidirectional bar chart to display the data. The ratings ‘a:not at all’ and ratings ‘better than a’ are displayed on the left and right sight of the vertical axis, respectively. Two achieve this separation the data set ‘matrix_3a’ is split into the two data sets ‘m1a’ (only a ratings) and ‘m2a’ (only ratings better than a) and the counts of data set ‘m1a’ are multiplicated with -1.

```
m1a<-matrix_3a %>% filter(rating == "rating_a") %>% mutate(count = count *-1)
m2a<-matrix_3a %>% filter(rating == "ratings_better_a")
```

Reunite the two data sets

```
matrix_4a <- rbind(m1a,m2a)

##    ID   Publication                rating             count
## 1   1   Björk et al., 2017         rating_a           -5
## 2   2   Dadvand et al. 2016        rating_a           -5
## 3   3   Orban et al. 2017          rating_a           -8
## 4   4   Reklatiene et al. 2014     rating_a           -6
## 5   5   Ruijsbroek et al. 2017     rating_a           -4
## 6   6   Stronegger et al. 2010     rating_a           -6
## 7   7   Triguero-Mas et al. 2015   rating_a           -5
## 8   1   Björk et al., 2017         ratings_better_a   9
## 9   2   Dadvand et al. 2016        ratings_better_a   9
## 10  3   Orban et al. 2017 ratings_better_a            5
## 11  4   Reklatiene et al. 2014     ratings_better_a   8
## 12  5   Ruijsbroek et al. 2017     ratings_better_a   9
## 13  6   Stronegger et al. 2010     ratings_better_a   8
## 14  7   Triguero-Mas et al. 2015   ratings_better_a   9
```

1.1.8 2b) Creation of plot_a

```
plot_a <-    ggplot(data = matrix_4a, aes(x = count, y = Publication, group = rating)) +
             geom_col(aes(fill = rating), width=0.5) +
             geom_vline(xintercept = 0) +
             scale_fill_manual(breaks=c("rating_a", "ratings_better_a") ,
                       values = c("#BCD2EE ", "#000080"),
                       labels=c("Rating a (not at all)", "Rating better than a (not at all)")) +
             geom_hline(yintercept = 0, color =c("black"))
```

Scale and Axes

```
plot_a <-    plot_a +
             labs(y = "", x = "Number of Criteria") +
```

```
                    scale_x_continuous(breaks=seq(-14, 14, 2),
                                       labels=c(14,12,10,8,6,4,2,0,2,4,6,8,10,12,14),
                                       limits = c(-14, 14))
```

Theme and legend

```
plot_a <-           plot_a +
                    theme_light() +
                    theme(legend.position = "bottom",
                             legend.title=element_blank(),
                             legend.text = element_text(size=12),
                             axis.text = element_text(size = 12))
```

Print plot and save as *.png*

```
plot_a

#ggsave("plot_1.png")
```

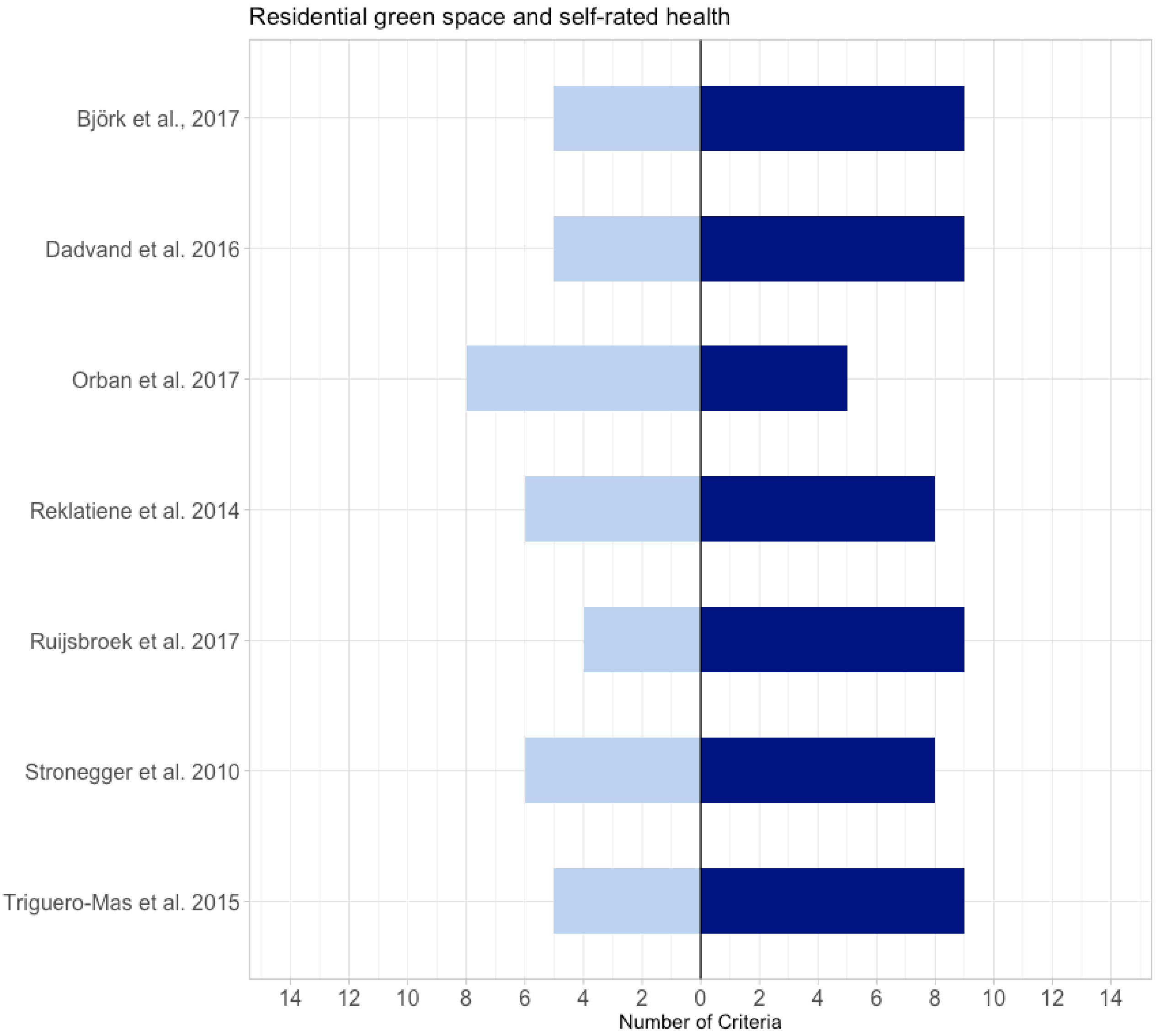

Supplementary figure 1: Number of criteria rated as better than a (not at all) per publication; Publications identified by the systematic review of Bolte et al. assessing sex/gender in the association between residential green space and self-rated health (*n* = 7)

1.1.9 4) Plot 2: Frequency of how often the different rating levels have been achieved for every criterion

In this section we describe how to create the bidirectional bar chart displaying the frequency of how often the different rating levels have been achieved for every criterion. It requires the data set 'matrix' that has been imported in section 1). To successfully apply the following syntax please load the packages *tydr*, *dplyr*, *ggplot* and *stringr*.

1.1.10 4.1) Transformation of the dataset

Create data set matrix_1b

Data set 'matrix_1b' is based on the original data set 'matrix'. It contains only contains the criteria and their ratings.

```
matrix_1b <- data.frame(matrix$C_1,matrix$C_2, matrix$C_3, matrix$C_4, matrix$C_5, matrix$C_6, matrix$C_7,
                        matrix$C_8, matrix$C_9, matrix$C_10, matrix$C_11, matrix$C_12, matrix$C_13,
                              matrix$C_14)
```

Transformation of matrix_1b

The data set 'matrix_2b' is based on the data set 'matrix_1b'. The function *gather* is used to change the form of a data set. Columns are transformed to rows. The column 'criteria' contains the different criteria of the matrix. The column 'ratings' now separates between the different rating levels.

```
matrix_2b <- gather(matrix_1b, key="criteria", value="ratings", 1:14)

##       criteria      ratings
## 1      matrix.C_1   a
## 2     matrix.C_1    a
## 3     matrix.C_1    a
## 4     matrix.C_1    a
## 5     matrix.C_1    a
## 6     matrix.C_1    a
## 7     matrix.C_1    a
## 8     matrix.C_2    a
## 9     matrix.C_2    a
## 10    matrix.C_2    a
## 11    matrix.C_2    a
## 12    matrix.C_2    a
## 13    matrix.C_2    a
## 14    matrix.C_2    a
## 15    matrix.C_3    c
## 16    matrix.C_3    c
## 17    matrix.C_3    b
## 18    matrix.C_3    c
## 19    matrix.C_3    c
## 20    matrix.C_3    d
```

New variable: Count number of ratings per criteria

The data set 'matrix_3b' contains a new variable 'n' that gives information about the frequency the different rating levels have been achieved per criteria.

```
matrix_3b <- matrix_2b %>%
               count(criteria, ratings)
```

New variable: level_count (number of rating levels per criteria)

Not every criterion consists of the same amount of rating levels. The variable 'level_count' contains the information on the number of rating levels per criteria.

```
matrix_3b <- mutate(matrix_3b, level_count = ifelse(criteria == "matrix.C_1" |
                                                    criteria == "matrix.C_2" |
                                                    criteria == "matrix.C_6" |
                                                    criteria == "matrix.C_7" |
                                                    criteria == "matrix.C_12", 2,
                                               ifelse(criteria == "matrix.C_4" |
                                                    criteria ==  "matrix.C_5" |
                                                    criteria ==  "matrix.C_8" |
                                                    criteria ==  "matrix.C_9" |
                                                    criteria ==  "matrix.C_10" |
                                                    criteria ==  "matrix.C_11" |
                                                    criteria ==  "matrix.C_14", 3, 4)))

##       criteria       ratings  n       level_count
## 1     matrix.C_1     a        7       2
## 2     matrix.C_10    a        7       3
## 3     matrix.C_11    c        7       3
## 4     matrix.C_12    b        7       2
## 5     matrix.C_13    c        3       4
## 6     matrix.C_13    d        4       4
## 7     matrix.C_14    a        1       3
## 8     matrix.C_14    b        3       3
## 9     matrix.C_14    c        3       3
## 10    matrix.C_2     a        7       2
## 11    matrix.C_3     b        1       4
## 12    matrix.C_3     c        5       4
## 13    matrix.C_3     d        1       4
## 14    matrix.C_4     a        3       3
## 15    matrix.C_4     c        4       3
## 16    matrix.C_5     a        2       3
## 17    matrix.C_5     c        5       3
## 18    matrix.C_6     a        7       2
## 19    matrix.C_7     a        1       2
## 20    matrix.C_7     b        4       2
```

New variable: level

To ensure that every rating level is displayed in the correct color, the variable 'level' is needed. It depends on the number of rating levels.

```
matrix_3b <- mutate(matrix_3b, level = ifelse(ratings == "a" , "l_1",
                                          ifelse(level_count == 3 & ratings == "b", "l_3",
                                          ifelse(level_count == 4 & ratings == "b", "l_2",
                                          ifelse(level_count == 4 & ratings == "c", "l_3", "l_4")))))
```

Preparation and sorting of the variables

For the next step the variable 'n' is transformed as numeric, the variable 'level' as factor and the variable 'criteria' as character variable. You might also sort the data set.

```
matrix_3b$n<-as.numeric(matrix_3b$n)
matrix_3b$level<-as.factor(matrix_3b$level)
matrix_3b$criteria<-as.character(matrix_3b$criteria)

matrix_3b$criteria <-  factor(matrix_3b$criteria, ordered = TRUE, levels = c( "matrix.C_14", "matrix.C_13",
                                        "matrix.C_12","matrix.C_11", "matrix.C_10", "matrix.C_9", "matrix.C_8",
```

```
                                             "matrix.C_7",   "matrix.C_6", "matrix.C_5", "matrix.C_4", "matrix.C_3",
                                             "matrix.C_2", "matrix.C_1"))

matrix_3b$level <-  factor(matrix_3b$level, ordered = TRUE, levels = c("l_4", "l_3","l_2", "l_1"))
```

Split data set

We choose a bidirectional bar chart to display the data. The ratings ‘a: not at all’ and ratings ‘better than a’ are displayed on the left and right sight of the vertical axis, respectively. Two achieve this separation the data set ‘matrix_3b’ is split into the two data sets ‘m1b’ (only a ratings) and ‘m2b’ (only ratings better than a) and the counts of data set ‘m1b’ are multiplicated with -1.

```
m1b<-matrix_3b %>% filter(level == "l_1") %>% mutate(n = n *-1)
m2b<-matrix_3b %>% filter(level == "l_2" | level== "l_3" | level=="l_4")
```

Reunit dataframe and delete ‘not applicable’

```
matrix_4b <- rbind(m1b,m2b)
matrix_5b<-matrix_4b[!(matrix_4b$ratings=="NA"),]
matrix_5b$criteria <- factor(matrix_5b$criteria, ordered = TRUE,
                                                      levels = c("matrix.C_14",
                                                                 "matrix.C_13",
                                                                 "matrix.C_12",
                                                                 "matrix.C_11",
                                                                 "matrix.C_10",
                                                                 "matrix.C_9",
                                                                 "matrix.C_8",
                                                                 "matrix.C_7",
                                                                 "matrix.C_6",
                                                                 "matrix.C_5",
                                                                 "matrix.C_4",
                                                                 "matrix.C_3",
                                                                 "matrix.C_2",
                                                                "matrix.C_1"))

##      criteria        ratings  n    level_count   level
## 1    matrix.C_1      a        -7   2             l_1
## 2    matrix.C_10     a        -7   3             l_1
## 3    matrix.C_14     a        -1   3             l_1
## 4    matrix.C_2      a        -7   2             l_1
## 5    matrix.C_4      a        -3   3             l_1
## 6    matrix.C_5      a        -2   3             l_1
## 7    matrix.C_6      a        -7   2             l_1
## 8    matrix.C_7      a        -1   2             l_1
## 9    matrix.C_9      a        -4   3             l_1
## 10   matrix.C_11     c        7    3             l_4
## 11   matrix.C_12     b        7    2             l_4
## 12   matrix.C_13     c        3    4             l_3
## 13   matrix.C_13     d        4    4             l_4
## 14   matrix.C_14     b        3    3             l_3
## 15   matrix.C_14     c        3    3             l_4
## 16   matrix.C_3      b        1    4             l_2
## 17   matrix.C_3      c        5    4             l_3
## 18   matrix.C_3      d        1    4             l_4
## 19   matrix.C_4      c        4    3             l_4
## 20   matrix.C_5      c        5    3             l_4
```

4.2) Creation of plot_b

```
plot_b <- ggplot(data = matrix_5b, aes(x = n, y = criteria, group = level)) +
          geom_col(aes(fill = level), width=0.5) +
          geom_vline(xintercept = 0) +
          geom_text(aes(label = ratings), colour = “white”, position = position_stack(vjust = 0.5)) +
          scale_fill_manual(breaks=c("l_1", "l_2", "l_3", "l_4") ,
                            values = c("#BCD2EE", "#87CEFA", "#1874CD ", "#000080")) +
          geom_hline(yintercept = 0, color =c("black"))
```

Add label

```
plot_b <- plot_b + scale_y_discrete( labels=c("matrix.C_1" = "1) Precise sex/gender terms used",
                                              "matrix.C_2" = "2) Sex/gender in the title",
                                              "matrix.C_3" = "3) Sex/gender in the abstract",
                                              "matrix.C_4" = "4) Sex/gender in the rationale",
                                              "matrix.C_5" = "5) Sex/gender in the objectives",
                                              "matrix.C_6" = "6) Sex/gender in the hypotheses",
                                              "matrix.C_7" = "7) Recruitment information described",
                                              "matrix.C_8" = "8) Sex/gender specific recruitment described",
                                              "matrix.C_9" = "9) Source of sex/gender information reported",
                                              "matrix.C_10" = "10) Sex/gender dimensions/ variability considered",
                                              "matrix.C_11" = "11) Sex/gender analysis reported",
                                              "matrix.C_12" = "12) Sex/gender distribution reported",
                                              "matrix.C_13" = "13) Sex/gender findings reported",
                                              "matrix.C_14" = "14) Sex/gender findings discussed"))
```

Scales

```
plot_b <- plot_b +  labs(y = "", x = "Number of publications") + scale_x_continuous(breaks=seq(-7, 7, 1),
                   labels=c("7","6","5","4 \nrating a \n(not at all)","3","2","1","0","1","2","3",
                            "4 \nrating better \nthan a (not at all)","5","6","7"))
```

Theme and Legend

```
plot_b <- plot_b +  theme_light() +
      theme(legend.position = "none", axis.text = element_text(size = 12))
```

Print plot and save as *.png*

```
plot_b

#ggsave("plot_2.png")
```

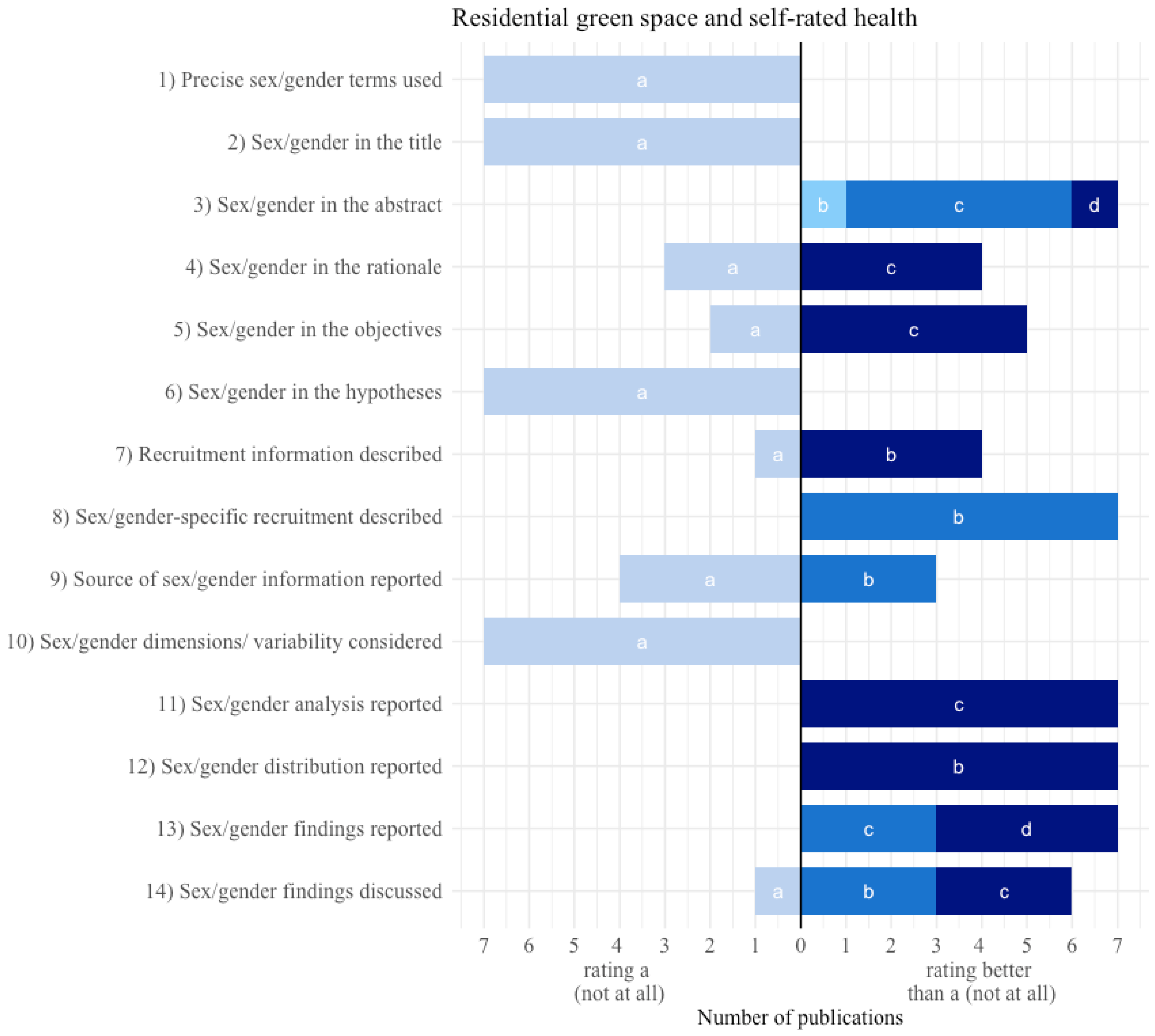

Supplementary figure 2: Number of publications with a specific rating of the 14 criteria to assess sex/gender consideration. Fulfilment of the evaluation criterion: a - not at all; b, c or d - to a certain extent depending on the specific criterion (for a detailed explanation see results section); publications identified by the systematic review of Bolte et al. assessing sex/gender in the association between residential green space and self-rated health (n = 7).
